# Proton versus photon craniospinal irradiation for adult medulloblastoma: A dosimetric, toxicity, and exploratory cost analysis

**DOI:** 10.1093/noajnl/vdae034

**Published:** 2024-03-08

**Authors:** William G Breen, Connie S Geno, Mark R Waddle, Jing Qian, William S Harmsen, Terry C Burns, Ugur T Sener, Michael W Ruff, Bryan J Neth, Joon H Uhm, David M Routman, Elizabeth Yan, Jon J Kruse, Nadia N Laack, Paul D Brown, Anita Mahajan

**Affiliations:** Department of Radiation Oncology, Mayo Clinic, Rochester, Minnesota, USA; Department of Radiation Oncology, Mayo Clinic, Rochester, Minnesota, USA; Department of Radiation Oncology, Mayo Clinic, Rochester, Minnesota, USA; Department of Radiation Oncology, Mayo Clinic, Rochester, Minnesota, USA; Department of Biomedical Statistics and Informatics, Mayo Clinic, Rochester, Minnesota, USA; Department of Neurologic Surgery, Mayo Clinic, Rochester, Minnesota, USA; Division of Medical Oncology, Mayo Clinic, Rochester, Minnesota, USA; Division of Medical Oncology, Mayo Clinic, Rochester, Minnesota, USA; Division of Medical Oncology, Mayo Clinic, Rochester, Minnesota, USA; Division of Medical Oncology, Mayo Clinic, Rochester, Minnesota, USA; Department of Radiation Oncology, Mayo Clinic, Rochester, Minnesota, USA; Department of Radiation Oncology, Mayo Clinic, Rochester, Minnesota, USA; Department of Radiation Oncology, Mayo Clinic, Rochester, Minnesota, USA; Department of Radiation Oncology, Mayo Clinic, Rochester, Minnesota, USA; Department of Radiation Oncology, Mayo Clinic, Rochester, Minnesota, USA; Department of Radiation Oncology, Mayo Clinic, Rochester, Minnesota, USA

**Keywords:** brain tumors, healthcare costs, medulloblastoma, proton therapy, radiotherapy

## Abstract

**Background:**

This study aimed to determine whether proton craniospinal irradiation (CSI) decreased the dose to normal tissue and resulted in less toxicity than photon CSI for adult patients.

**Methods:**

This single-institution retrospective analyzed differences in radiation doses, acute toxicity, and cost between proton and CSI for adult medulloblastoma patients.

**Results:**

Of 39 total patients, 20 were treated with photon CSI prior to 2015, and 19 were treated with proton CSI thereafter. Median age was 28 years (range 18–66). The molecular subtype was most commonly sonic hedgehog (68%). Patients most commonly received 36 Gy CSI in 20 fractions with a boost to 54–55.8 Gy (92%). Proton CSI delivered significantly lower mean doses to cochleae, lacrimal glands, lens, parotid glands, pharyngeal constrictors, esophagus, lungs, liver, and skin (all *P* < .001). Patients receiving proton CSI had significantly lower rates of acute dysphagia of any grade (5% versus 35%, *P* = .044) and decreased median weight loss during radiation (+1.0 versus –2.8 kg, *P* = .011). Weight loss was associated with acute hospitalization (*P* = .009). Median follow-up was 2.9 and 12.9 years for proton and photon patients, respectively, limiting late toxicity and outcome comparisons. At the last follow-up, 5 photon patients had died (2 of progressive disease, 3 without recurrence ages 41–63) and 21% had experienced major cardiovascular events. At 10 years, 89% were alive and 82% were recurrence free.

**Conclusions:**

This study demonstrates dosimetric improvements with proton CSI, potentially leading to decreased acute toxicity including dysphagia and weight loss during treatment.

**Key Points**

- Proton craniospinal irradiation (CSI) significantly decreases radiation dose to organs compared to standard photon radiation.
- Proton CSI was associated with less dysphagia and weight loss than photon CSI. Cost analysis did not show that protons increased cost.

**Importance of the Study**

Medulloblastoma is a rare disease in adults with limited high-level data to guide management. While proton craniospinal irradiation (CSI) is utilized at some centers when available, the specific benefits of proton CSI over standard photon CSI in an adult population have not been rigorously described. This retrospective analysis demonstrates that proton CSI decreases radiation dose to many organs, including the esophagus and pharyngeal constrictors. Accordingly, proton CSI is associated with less acute dysphagia and weight loss than photon CSI, with weight loss during treatment significantly associated with the risk of hospitalization. This analysis includes long-term adverse events observed in patients receiving photon CSI after a median follow-up of greater than 12 years. This manuscript also contains a novel cost comparison between modalities, demonstrating that proton CSI is not associated with increased costs compared to photon CSI.

While medulloblastoma is one of the most common malignant brain tumors of childhood, it is also diagnosed in adults, though much less frequently.^1,2^ Because of the rarity of adult medulloblastoma, despite potential biologic differences between adult and pediatric medulloblastoma, its treatment typically follows the multidisciplinary paradigm established in pediatric patients: maximal safe surgical resection, craniospinal irradiation (CSI) with a boost to the primary site, and chemotherapy.^3–5^

CSI is an essential component of treatment for medulloblastoma but has been associated with significant acute and late adverse effects and impacts on quality of life.^6^ Traditionally, CSI has been delivered with photon-based radiotherapy.^7^ More recently, proton CSI has been used when available to reduce the radiation dose to normal tissue in an effort to reduce acute and late toxicities.^8,9^ In pediatric patients, proton CSI appears to provide equivalent oncologic outcomes while potentially decreasing the risk of acute and late radiation effects and has become a standard treatment.^10–13^ While proton CSI will be incorporated in cooperative group clinical trials for adult medulloblastoma, currently there are no prospective data demonstrating the benefit of proton CSI over photon CSI in adult patients.^5,14^ Although prior retrospective analyses from a single institution have shown dosimetric and toxicity benefits to CSI, it is unknown whether these findings are generalizable, and what impact proton therapy may have on tolerance of adjuvant chemotherapy and healthcare and associated costs.^8,9^

In this study, we reviewed the medical records of adult medulloblastoma patients receiving photon and proton CSI and compared radiation dose to normal tissue as well as acute toxicity. Exploratory descriptions of late toxicity, outcomes, and cost analyses are also provided.

## Methods

This retrospective study was approved by the Institutional Review Board (IRB) and conducted in accordance with The Code of Ethics of the World Medical Association (Declaration of Helsinki).

### Patients

A radiation-specific institutional database which included prospectively collected physician-assessed toxicities during treatment and in follow-up was utilized to identify adult patients (age 18+ at the time of diagnosis) who were treated with CSI for pathologically confirmed medulloblastoma from 2000 to 2020. Patients who received only palliative or focal radiotherapy, or whose radiotherapy records were unavailable for review were excluded.

### Treatment

Patients were treated at the discretion of their treating physicians and according to the standard of care at the time of treatment. Radiation simulation, treatment volumes, planning, and dose fractionation were at the discretion of the treating radiation oncologist. Photon plans were either 3-dimensional conformal radiotherapy (3DCRT) or intensity-modulated radiotherapy (IMRT). Photon plans used either posterior spine fields or posterior oblique spine fields with dynamic multileaf collimators to create feathered junctions. Proton patients were treated with scanning-beam proton therapy, using posterior spinal fields (with the number depending on patient height), and typically an anteriorly tipped vertex (headbutt) and posteriorly tipped vertex (reverse headbutt) or posterior cranial field. Proton plans were robustly optimized with overlapping fields to create a homogenous dose distribution at the junctions between fields. Monte Carlo-based biologic dose was calculated to evaluate and minimize hotspots in the spinal cord and brainstem.^15,16^

Dosimetric data was obtained from the treatment planning system, with older photon treatment records pulled from the prior treatment planning system. In older plans where specific organs at risk dose statistics were not available, these structures were retrospectively contoured, and doses were calculated whenever possible.

### Patient Assessments

During treatment, patients were assessed once per week for acute toxicities. Following treatment, patients underwent routine clinical surveillance with diagnostic imaging, typically at intervals of 3–6 months. Toxicities were in some cases prospectively assessed, recorded, and attributed by the treating physician, and all cases were reviewed retrospectively. Acute toxicities were defined as those occurring during RT or within 90 days of treatment completion. Late toxicities occurred greater than 90 days after completing radiotherapy. Blood counts including hemoglobin, white blood cells (WBC), and platelets were recorded before and after radiotherapy. Survival and progression outcomes were assessed retrospectively.

### Statistical Analyses

Summary statistics were generated for all patient and treatment variables. Paired *t*-tests were performed to determine whether there was a significant difference in dosimetric variables between proton and photon CSI. Univariate logistic regression models were generated to assess the association of patient and treatment variables with acute toxicities. Spearman rank correlation was performed to assess the association of dosimetric variables with changes in continuous dependent variables including hemoglobin, platelets, WBC, weight (kg), and BMI. Overall survival and disease-free survival were calculated from the start of CSI using the Kaplan–Meier method.

### Cost Analysis

An exploratory cost analysis was performed for patients with cost data available, in order to compare proton and photon CSI. Patients were treated in the United States. Standardized costs were obtained and validated for all patients through our institutional cost data warehouse.^17^ Medicare reimbursement was assigned to all professional services, the appropriate Medicare Cost Report cost-to-charge ratios were applied to charges for all hospital billed services, and all resulting costs were adjusted upwards due to inflation from the year of treatment per the GDP Implicit Price Deflator.

Total costs per patient were collected from 3 months prior to until 6 months following radiation initiation, consistent with prior methodology. These were then characterized as radiation-related or as other costs. Radiation costs were subdivided into radiation treatment delivery, radiation planning, simulation and management, or other radiation costs. Nonradiation costs were categorized as hospital services, procedures, imaging, tests/labs, outpatient visits, oncology/chemotherapy, or others based on appropriate billing codes and billing locations. To compare average costs between proton and photon CSI, the Mann–Whitney *U* test was used due to the non-normal distribution of treatment costs.

## Results

Thirty-nine patients met inclusion criteria for this study, including 20 treated with photon CSI prior to 2015, and 19 treated with proton therapy thereafter (Table 1). Among those receiving photon CSI, 11 received IMRT and 9 received 3DCRT. For the entire cohort, the median age was 28 years (range 18–66). The molecular subtype was most commonly sonic hedgehog (68%). Patients most commonly received 36 Gy in 20 fractions of CSI (72%) with a primary tumor/posterior fossa boost to 54–55.8 Gy (92%). While patients typically underwent maximally safe resection, followed by adjuvant radiotherapy and then chemotherapy, chemotherapy was delivered prior to radiation in 5 patients (4 photon (20%) and 1 proton (5%)).

**Table 1.** Patient and Treatment Characteristics

		Photon *N* = 20	Proton *N* = 19
Age (years)	Median (IQR)	31.5 (27.1–42.1)	25.7 (20.6–30.5)
Sex	Female	7	8
	Male	13	11
Weight (kg)	Median (IQR)	81 (70–91)	82 (71–102)
Histology	Classical	5 (46%)	7 (64%)
	Desmoplastic	5 (46%)	4 (36%)
	Anaplastic	1 (9.1%)	
	Not specified/missing	*N* = 9	*N* = 8
Subgroup	WNT	1 (17%)	2 (11%)
	SHH	5 (83%)	12 (63%)
	Group 4	0 (0%)	1 (5%)
	Non-WNT/SHH	0 (0%)	4 (21%)
	Missing	14	
Resection Extent	GTR	11 (55%)	13 (68%)
	Near-GTR	3 (15%)	2 (11%)
	STR	3 (15%)	3 (16%)
	Biopsy only	3 (15%)	1 (5%)
M stage	0	16 (80%)	13 (68%)
	1	0 (0%)	0 (0%)
	2	0 (0%)	4 (21%)
	3	3 (15%)	2 (11%)
	4	1 (5%)	0 (0%)
CSI Dose (Gy)	Median (range)	36 (23.4–39.6)	36 (23.4–36)
CSI Fractions	Median (range)	20 (13–22)	20 (13–20)
Boost Dose	Median (range)	19.8 (14.4–32.4)	19.8 (16.2–30.6)
Total Dose	Median (range)	55.8 (54–55.8)	54 (50.4–57.6)

**Figure 1.**
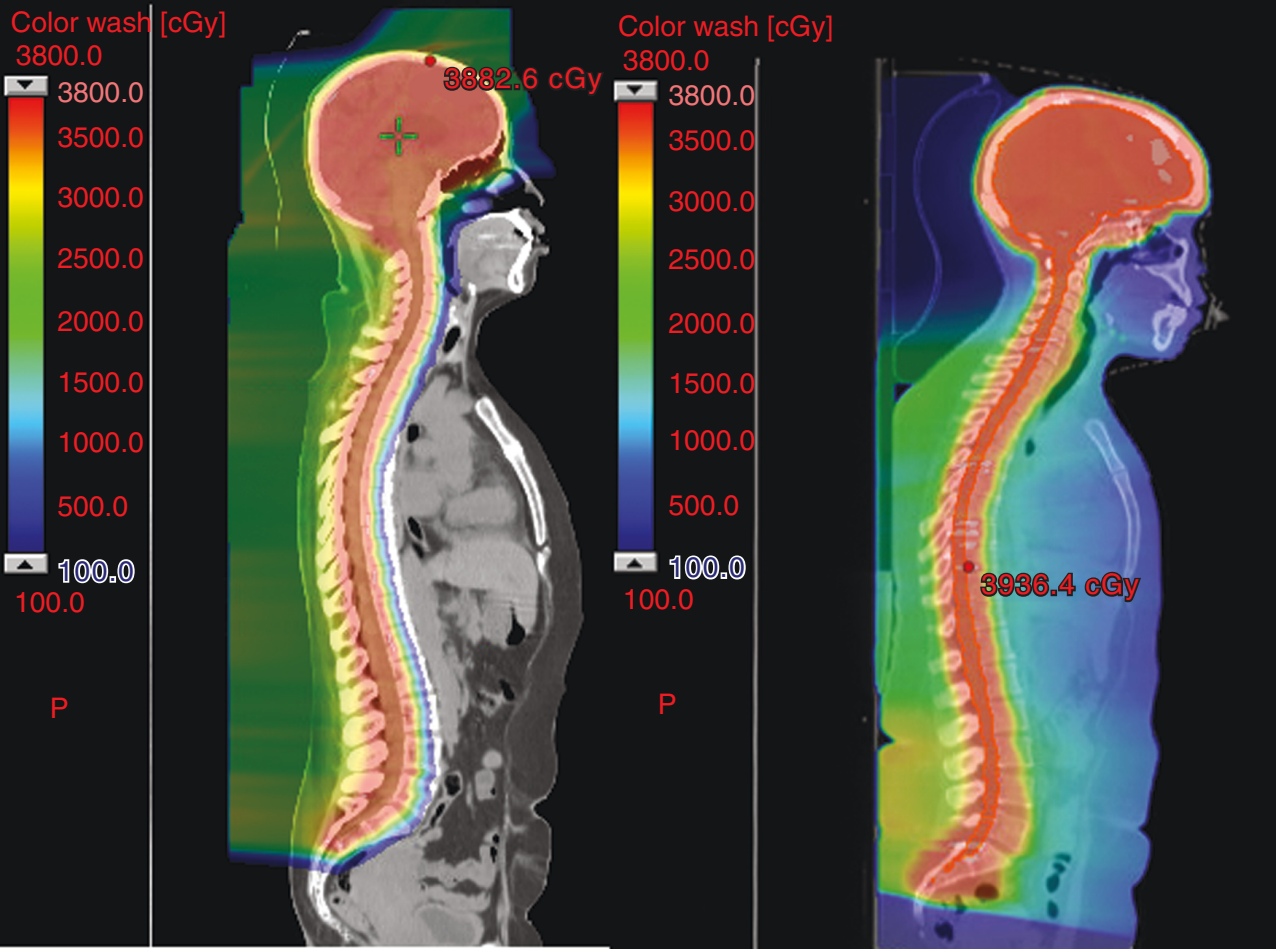
Comparison of radiation dose between patients receiving proton CSI (left) and photon CSI (right), demonstrating increased dose spill anteriorly into the esophagus, thorax, abdomen, and pelvis with photon CSI.

Proton CSI delivered significantly lower radiation doses to cochleae, lacrimal glands, lens, parotid glands, pharyngeal constrictors, esophagus, lungs, liver, and skin (Table 2 and Figure 1). The mean heart dose was decreased from 14.4 Gy in photon patients to 0 Gy in proton patients (*P* < .01).

**Table 2.** Dosimetric Statistics for Proton versus Photon Craniospinal Irradiation (CSI)

Dosimetric Variable	Photon Median (Gy)	Proton Median (Gy)	*P*-value
Right cochlea mean	43.6	31.5	<.001
Left cochlea mean	44.1	31.7	.002
Right lacrimal gland mean	35.9	8.4	<.001
Left lacrimal gland mean	36.5	8.2	<.001
Right lens max	29.7	2.1	<.001
Left lens max	27.2	2.3	<.001
Parotid (total) mean	25.5	3.1	<.001
Liver mean	7.0	0.1	<.001
Kidney V12Gy (total)	12.6%	0.5%	<.001
Lung (total) mean	6.9	1.4	<.001
Lung V20 Gy (total)	7.2%	1.9%	<.001
Heart mean	14.4	0.0	<.001
Esophagus mean	29.3	1.5	<.001
Pharyngeal constrictors mean	15.2	6.2	<.001
Skin max	50.9	38.2	<.001

Patients receiving photon CSI had significantly higher rates of acute dysphagia of any grade (35% versus 5%, *P* = .044) and greater median weight loss during radiation (–2.8 kg versus +1.0 kg, *P* = .011). Maximum esophagus dose was significantly associated with greater weight loss (*P* = .0499). Acute hospitalization was associated with increased weight loss during radiotherapy (*P* = .009). Three photon and no proton patients were hospitalized during the acute period. There were no other significant differences in acute toxicities between proton and photon CSI, including nausea, vomiting, fatigue, need for IV fluids, need for transfusion, and admission to a rehabilitation center, and no significant associations between dosimetric parameters and these acute toxicities.

There were no significant differences in changes in blood counts between proton and photon CSI, though the magnitude of change in platelets for patients receiving photon CSI appeared higher. Platelets dropped by a median of 70.5 and 17.5 platelets per microliter for photon and proton CSI, respectively (*P* = .080). Hemoglobin dropped by a median of 0.5 and 0.6 g/dL for photon and proton CSI, respectively (*P* = .387). White blood cells dropped by an average of 2.0 and 2.8, respectively (*P* = .7984).

Median follow-up was 12.9 and 2.9 years for photon and proton patients, respectively, limiting late toxicity and outcome comparisons. At 10 years, 89% were alive and 82% were free of recurrence. Five photon CSI patients had died at the last follow-up (2 of progressive disease and3 without recurrence ages 41–63) compared to 0 proton patients. Three photon and 1 proton patient had experienced progression. Late major cardiovascular events were seen in 21% and 0% of photon and proton patients, respectively. One photon patient had a myocardial infarction and heart failure, 2 had strokes, and 1 had SMART syndrome. Five patients (25%) who received photon CSI had late endocrine dysfunction. Five photons (25%) and 1 proton patient (5%) had late fatigue.

Cost data was available from the cost data warehouse for 18 patients, including 12 photon and 6 proton patients, limiting the power of cost analyses (Supplementary Table 1). There was no significant difference in total radiation costs ($25 110 for protons versus $23 650 for photons, *P* = .40), hospital services ($23 420 for protons versus $32 630 for photons, *P* = .67), or total 6-month costs ($72 413 for protons versus $83 568 for photons, *P* = .71).

## Discussion

In this retrospective analysis of adult medulloblastoma patients receiving proton or photon CSI, proton CSI was associated with significantly lower doses to normal tissues including esophagus and pharyngeal constrictors, and thereafter proton CSI patients experienced less acute dysphagia and weight loss. Weight loss was associated with the risk of acute hospitalization, leading to the presumption that the reduced esophagus and constrictor dose from proton CSI may reduce hospitalizations related to dysphagia.

In an analysis of adult medulloblastoma patients receiving proton or photon CSI at MD Anderson Cancer Center, Brown and colleagues also found increased esophagitis and weight loss with photon CSI compared to proton CSI.^8^ While the analysis by Brown et al. and the present analysis contain only 40 and 39 patients respectively, it lends strength to this finding that it was statistically significant in these independent datasets from separate institutions. In contrast to the study by Brown et al., this analysis also quantitatively describes the decrease in radiation dose with proton CSI to the esophagus and pharyngeal constrictors (in addition to other organs at risk) to explain this finding further mechanistically.

As evidenced by the high rate of progression-free and overall survival at 10 years demonstrated in this analysis, consideration of late toxicities from therapy and long-term quality of life is critical in this population.^6^ This analysis demonstrates reductions in doses to the heart and lungs with proton CSI compared to photon CSI which may become clinically important with further follow-up given well-established data showing dose relationships with toxicity in these organs.^9,18,19^ In this analysis, there were several patients in the photon CSI cohort with late nononcologic death, and over 20% of the photon CSI patients had a major late vascular event. Considering the significant decrease in radiation dose to the heart with protons demonstrated in this analysis (heart mean dose 14 Gy for photons versus 0 Gy for protons), there is hope that longer follow-up in the proton cohort will demonstrate lower rates of late cardiovascular events.

Molecular subtypes have been elucidated from the study of pediatric medulloblastoma, and carry significant prognostic value and increasingly guide treatment strategies in that population.^20,21^ However, multiple analyses indicate that adult medulloblastoma may be biologically and clinically varied from pediatric cases, and recent data indicates that molecular subtypes may carry less prognostic value in adults.^22–25^ Adult populations may have a higher proportion of patients with sonic hedgehog (SHH) molecular subtype, who could theoretically benefit from Smoothened (SMO) inhibitors.^26,27^ Data on the effectiveness of these drugs in adult medulloblastoma patients is awaited, and further elucidation of the molecular underpinnings and their therapeutic implications in adult medulloblastoma are being planned.^5,14^

We attempted to perform a cost comparison between proton and photon CSI, however, this was limited as just under half of the patients had cost data available. No significant differences were found between proton and photon CSI costs, with a total 6-months cost of $72 413 for protons versus $83 568 for photons. The numeric difference between these totals was driven by the difference in costs of hospitalization during treatment, at $23 420 for protons versus $32 630 for photons.

This small, relatively heterogeneous retrospective study is subject to all the limitations of such studies. Proton and photon patients were treated in different eras which complicates the comparison. Importantly, the cohorts have considerably different follow-up durations limiting the ability to credibly compare late toxicities or oncologic outcomes between proton and photon CSI in this analysis. With modern photon CSI techniques including volumetric arc therapy (VMAT), differences between proton and photon CSI may be less pronounced. An analysis of pediatric medulloblastoma patients by Liu et al. demonstrated higher lymphocyte counts in patients receiving proton CSI; we were not able to demonstrate significant differences in changes in blood counts between modalities in this retrospective study potentially due to small sample size and heterogenous systemic therapy regimens and timing.^12^

## Supplementary Material

vdae034_suppl_Supplementary_Table_S1
