# Supplementary material for: Proton versus photon craniospinal irradiation for adult medulloblastoma: A dosimetric, toxicity, and exploratory cost analysis
Source: Neurooncol Adv. 2024 Mar 8;6(1):vdae034. doi: 10.1093/noajnl/vdae034 (PMC10976906; doi:10.1093/noajnl/vdae034)
Supplement: vdae034_suppl_Supplementary_Table_S1 [file vdae034_suppl_supplementary_table_s1.docx]

| Supplemental Table 1: Selected costs for proton versus photon CSI in patients with available data | | | | | |
| --- | --- | --- | --- | --- | --- |
|  | Proton |  | Photon |  |  |
|  | N=6 |  | N=12 |  |  |
| 6-month Costs | Mean | Standard deviation | Mean | Standard deviation | p-value |
| Total Cost | $72,413 | $43,409 | $83,568 | $62,380 | 0.71 |
| Total Radiation Costs | $25,110 | $3,352 | $23,650 | $5,394 | 0.40 |
| Oncology/Chemotherapy | $1,795 | $1,657 | $1,307 | $1,351 | 0.64 |
| Hospital Services | $23,420 | $27,456 | $32,630 | $48,067 | 0.67 |
